# Six to seven times less likely to be physically active: Low self-determination and basic psychological needs support in women with endometriosis—A cluster analysis

**DOI:** 10.1371/journal.pone.0330446

**Published:** 2025-08-26

**Authors:** Clarissa Medeiros da Luz, Thiago Sousa Matias, Rafaella Russi de Paula, Juliana Araujo, Francielle Conceição Nascimento, Ygor Vieira de Oliveira, Esdras Camargos, Cristiano Denoni Freitas, Manuela Karloh

**Affiliations:** 1 Physical Therapy Graduate Program, Santa Catarina State University (UDESC), Florianópolis, Brazil; 2 Center for Assistance, Education and Research in Women’s Health (NuSIM), Santa Catarina State University (UDESC), Florianópolis, Brazil; 3 Department of Physical Education, School of Sports, Graduate Program in Physical Education, Graduate Program in Public Health, Federal University of Santa Catarina (UFSC), Florianópolis, Brazil; 4 Graduate Program in Human Movement Science, Center for Health Sciences and Sport (CEFID), Santa Catarina State University (UDESC), Florianópolis, Brazil; 5 Center for Assistance, Teaching and Research in Pulmonary Rehabilitation. Center for Health Sciences and Sport (CEFID), Santa Catarina State University (UDESC), Florianópolis, Brazil; 6 Surgeon in the Deep Infiltrating Endometriosis Service, Maternidade Carmela Dutra, Santa Catarina State Department of Health, Florianópolis, Brazil; University of Tartu, ESTONIA

## Abstract

**Purpose:**

To explore how clusters of motivational regulations, self-determination, and basic psychological needs (BPNs) relate to physical exercise participation in women with endometriosis.

**Methods:**

This cross-sectional online study included women aged ≥ 18 years with endometriosis. The Behavioral Regulation in Exercise Questionnaire-3 (BREQ-3) was used to assess motivational regulations and the Basic Psychological Needs in Exercise Scale (BPNES) to evaluate BPNs—autonomy (sense of choice), competence (confidence in ability), and relatedness (sense of connection and belonging)—followed by a two-step cluster analysis to identify motivational profiles. Associations with physical activity were tested using logistic regression (adjusted for pain score, education level, skin color, and marital status).

**Results:**

A total of 508 women (mean age = 34 ± 7 years) participated in the study. Women in the more controlled behavior cluster (OR = 6.14; 95% CI = 3.72–10.1) and in the BPN more thwarted cluster (OR = 7.61; 95% CI = 4.25–12.8) were significantly less likely to engage in physical activity compared to those in the more autonomous behavior — driven by personal interest — and in the more BPN satisfied cluster.

**Conclusions:**

Women with endometriosis with diminished self-determination and low BPNs support profile were six to seven times less likely to participate in physical activity, highlighting the need for tailored interventions to enhance motivation and exercise adherence.

## Introduction

Endometriosis is a chronic gynecological condition affecting approximately 5–15% of women of reproductive age [1]. This multifaceted condition presents with heterogeneous, often concurrent manifestations, resulting in a spectrum of debilitating symptoms, including severe dysmenorrhea, dyspareunia, dyschezia, hematochezia, tenesmus, diarrhea, chronic fatigue, infertility and more. [2–4].

Physical activity has been increasingly recommended as a conservative treatment for managing endometriosis due to its potential anti-inflammatory benefits, pain reduction, physical functionality, and ability to cope with stress improvement [5–8]. Women with endometriosis seem to engage less in physical activity compared with healthy controls. The limited engagement may be justified by the increased pain sensitivity, fear and avoidance behaviors, fatigue, and reduced social support [9]. Even in other situations where physical activity may play an important role in the management of symptoms from different diseases, individuals often do not maintain the habit, leading to disengagement over time [10–12].

Motivation has been widely studied concerning its role in adopting and maintaining healthy behaviors (e.g., physical activity) [13]. One of the most applied approaches to human motivation [14] is the self-determination theory (SDT), which explains why individuals choose to engage in and sustain behaviors, such as physical activities or rehabilitation programs, by examining the role of social contexts in fostering participation.

According to the SDT, the well-being and functioning of individuals are related to the fulfillment of three basic psychological needs (BPN): autonomy (sense of choice, willing or self-endorsement of your actions), competence (sense of being able and confident to act [or succeed] in your actions), and relatedness (sense of feeling belonged, socially connected, and cared for by others). SDT distinguishes motivation into autonomous (intrinsic, integrated, and identified regulations), driven by personal enjoyment and valuing, and controlled (introjected and external regulations), driven by obligation, reward, internal or external pressures; alongside amotivation, reflecting a lack of intention to act.

In the context of endometriosis, where chronic pain, fatigue, and emotional distress are common [6,15], these psychological needs may be particularly difficult to fulfill, making women more vulnerable to controlled motivation and amotivation. SDT provides a relevant framework to address these challenges by promoting autonomy-supportive environments and fostering more self-determined forms of motivation [16].

Evidence supports that autonomous motivation positively correlates with increased physical activity and overall well-being. However, controlled motivation and amotivation are associated with unhealthy behaviors, and may lead to disengagement and reduce overall well-being [14,16–20]. The process of changing behavior relies on the perception of the individual on BPN fulfillment and recognition of autonomous reasons to act. Therefore, interventions based on the SDT seek to enhance autonomous motivation by supporting BPN, and avoiding its frustration [21].

Despite the contributions of SDT across several populations and health domains [17,22], its application to physical activity motivation in women with endometriosis remains unexplored. This is a critical gap, as women with endometriosis face distinct psychological and physical barriers [9] to exercise participation compared to other population. Understanding their motivational profiles may provide tailored insights into overcoming these barriers and designing more effective physical activity interventions.

To date, SDT has only been applied to explore sexual motivation in couples coping with endometriosis [23] leaving its role in promoting physical activity motivation unexplored in this population. Beyond this limited scope, SDT has been effectively applied to other chronic pain populations, offering relevant insights for endometriosis management. For instance, more self- determined forms of motivation were associated with improved exercise behaviors in women with breast cancer, even in the presence of pain and fatigue [24]. Additionally, mindfulness-based interventions, aligned with SDT’s emphasis on psychological well-being, have shown promising effects on attention and parasympathetic regulation in women with endometriosis [25]. Peer support interventions for chronic pain management also appear to facilitate the satisfaction of BPNs, promoting self-management behaviors [26]. Furthermore, SDT-informed exercise programs have been found to improve disability and quality of life in individuals with chronic low back pain [27]. Collectively, this evidence underscores SDT’s potential to help overcome exercise barriers in chronic pain conditions such as endometriosis, even in the context of persistent pain and functional limitations.

Given that physical activity is essential for health promotion and plays a key role in managing symptoms of endometriosis [8], it is essential to support women with endometriosis in adopting and sustaining healthy behaviors. Thus, investigating the motivational profile is important to understand their likelihood of engaging in and maintaining physical activities. Deeper knowledge of motivation enables healthcare professionals to design strategies that facilitate the internalization of these behaviors, fostering more self-determined behavior forms of motivation and promoting long-term adherence to physical activity [13,17]. Therefore, this study aimed to investigate the association between clusters of motivational regulations, self-determination, basic psychological needs, and physical activity participation in women with endometriosis.

## Materials and methods

This cross-sectional study recruited women aged ≥ 18 years who self-reported a diagnosis of endometriosis, using convenience sampling via social media. Self-reported diagnosis was defined as participants declaring they had endometriosis without the study requiring formal confirmation through medical records, diagnostic imaging, or surgical findings. It was assumed that participants, by reporting a history of treatment for endometriosis (with or without specific medication or lesion-removal surgery), had likely received a medical diagnosis at some point. The snowball sampling method [28] was incorporated into social media by asking initial participants to disclose the invitation to other eligible women.

The recruitment period for this study began on 09/09/2021 and was concluded on 27/06/2023. All participants were thoroughly informed about the research and provided their consent via an electronic form. They had the opportunity to read the Informed Consent Form, and by clicking on the option ‘I acknowledge and agree to participate in the study’ and entering their names, they officially confirmed their participation.

The form (made on Microsoft Forms) included the medical history, scales, and questionnaires and was disclosed via messaging apps (WhatsApp and similar), websites, and social media. The form could be filled out using mobile phones, tablets, computers, or other devices with internet access. Duplicated or incomplete questionnaire responses were excluded from the analysis. Imputation was not performed due to the minimal rate of missing data.

All procedures of the study were performed in compliance with relevant laws and institutional guidelines and received approval from the research ethics committee of Santa Catarina State University (CAEE:25825819.5.0000.0118) and of Santa Catarina State Department of Health (CAEE:25825819.5.3001.0115). The sample was characterized by considering sociodemographic, clinical, and variables related to physical activity. The localization of endometriosis was divided into compartments: anterior compartment (bladder); posterior compartment (ovaries, intestine, uterosacral ligament, and vaginal vault); or distant organs (appendix, stomach, small intestine, diaphragm, and lungs).

### Behavioral Regulation in Exercise Questionnaire-3 (BREQ-3)

The Behavioral Regulation in Exercise Questionnaire-3 (BREQ-3) was used to assess motivational regulations in exercise. This instrument was validated for Brazilian Portuguese [29] and no major cultural modifications were required during the adaptation of the BREQ-3 for Brazilian adults. This validation included 470 women (45% of the sample) and confirmed factorial invariance across sexes, supporting the BREQ-3’s validity for Brazilian women. Most participants were young adults (21–30 years; 49.4%), reinforcing its applicability in young and middle-aged physically active women [29]. It comprises 23 questions with Likert responses ranging from 0 (not true for me at all) to 4 (very true for me). Scores were calculated according to the items assigned in each regulation: amotivation [(items 2 + 8 + 14 + 20)/4], external regulation [(items 6 + 12 + 18 + 23)/4], introjected regulation [(items 4 + 10 + 16)/3], identified regulation [(items 1 + 7 + 13 + 19)/4], integrated regulation [(items 5 + 11 + 17 + 22)/4], and intrinsic regulation [(items 3 + 9 + 15 + 21)/4]. Furthermore, these motivational regulations enabled the analysis of the Self-Determination Index – SDI (also called Relative Autonomy Index – RAI), in which positive weights are assigned for autonomous subscales and negative weights for those less self-determined. The SDI was calculated by the equation: SDI = (−3 x amotivation) + (−2 x external regulation) + (−1 x introjected regulation) + (1 x identified regulation) + (2 x integrated regulation) + (3 x intrinsic regulation) [30].

### Basic Psychological Needs in Exercise Scale (BPNES)

The fulfillment of BPN in exercise was assessed using the Basic Psychological Needs in Exercise Scale (BPNES). The validity and reliability of the Brazilian Portuguese version of the BPNEs have been established in a population with chronic respiratory diseases. No cultural modification were required during translation and adaption process [31]. Comparable reliability and construct validity were observed in women with endometriosis (data unpublished) [32]. This scale encompasses 11 items divided into three needs: autonomy, competence, and relatedness. Responses were provided on a 5-point Likert scale (1 = strongly disagree to 5 = strongly agree). The score was calculated by the mean of the responses to the items: autonomy [(questions 2 + 5 + 8 + 11)/4], competence [(questions 1 + 3 + 6 + 9)/4], and relatedness [(questions 4 + 7 + 10)/3]. High scores indicated increased fulfillment of BPN [33].

### Statistical analyses

Analyses were performed using SPSS (version 20.0), while GraphPad Prism (version 10.0) was used to create the figures. A two-step cluster analysis was performed, and two models were arranged. Seven variables were considered in the motivational regulations and self-determination model: self-determination index, amotivation, external regulation, introjected regulation, identified regulation, integrated regulation, and intrinsic regulation scores. In the BPN model, three variables were input: autonomy, competence, and relatedness scores. The two-step cluster procedure was chosen due to its ability to provide valuable interpretability while also maintaining a strong data-driven approach. The number of clusters was determined based on a combination of objective criteria, rather than relying on an arbitrary selection process. Specifically, we considered a low Schwarz’s Bayesian Criterion (BIC), a high ratio of distance measures, and significant changes in BIC, all of which were aligned with theoretical reasoning. This method revealed natural groupings within the sample. Additionally, continuous variables were included in both models, ensuring a comprehensive and accurate clustering process.

The quality of the cluster solution in the total data set was analyzed by silhouette coefficient indicating cohesion and separation (the silhouette ranges from − 1 to + 1; a high value indicates that the object is well matched to its cluster and poorly matched to neighboring clusters, with values closer to 1 indicating better separation and cohesion). The relative importance of each variable in the model was also observed. The predictor importance showed the relative importance of each variable in estimating the model and ranged from 0 to 1 (values close to 1 mean relatively more important). Multinomial logistic regression (crude and adjusted) was used to test the association between the identified clusters and physical activity participation, and results were expressed in odds ratio (OR) and 95% confidence intervals (CI).

The ‘more autonomous behavior’ cluster was the reference in the motivational regulations and self-determination model, and the ‘Basic psychological needs more satisfied’ cluster was the reference in the BPN model. Analyses were adjusted for pain score, education level, skin color, and marital status. Statistical significance was set at p < 0.05.

## Results

### Sample characteristics

A total of 534 women were initially assessed; 20 individuals were excluded due to incomplete electronic forms, while six duplicated responses were excluded from the analysis. Therefore, 508 women (mean age = 34 ± 7 years) participated in the study (Table 1).

**Table 1:** Sociodemographic characteristics and clinical aspects of the sample.

	n (%)	95% CI
**Skin color**		
White	313 (61.6)	57.3 to 65.9
Brown	124 (24.4)	20.7 to 28.2
Black	59 (11.6)	9.0 to 14.4
Yellow	10 (2.0)	1.0 to 3.2
Indigenous	2 (0.4)	0.05 to 1.4
**Education level**		
Complete higher education	285 (56.1)	51.7 to 60.4
Incomplete higher education	99 (19.5)	16.2 to 23.0
High school	105 (20.7)	17.3 to 24.3
Complete middle school	12 (2.4)	1.3 to 3.7
Incomplete middle school	7 (1.4)	0.6 to 2.5
**Marital status**		
Married or registered partnership	308 (60.6)	56.3 to 64.9
Single	172 (33.9)	29.8 to 38.2
Divorced or separated	25 (4.9)	3.2 to 6.8
Widowed	3 (0.6)	0.1 to 1.3
**Time to diagnose endometriosis**		
More than five years	271 (53.3)	49.0 to 57.6
Few months	91 (17.9)	14.7 to 21.3
2 to 3 years	74 (14.6)	11.6 to 17.8
One year	42 (8.3)	6.0 to 10.8
3 to 4 years	30 (5.9)	4.0 to 8.0
**Location of endometriosis**		
Posterior compartment	305 (60.0)	55.8 to 64.1
Other location or unknown	89 (17.5)	14.3 to 21.0
Anterior and posterior compartment	83 (16.3)	13.2 to 19.7
Posterior compartment and distant organs	14 (2.8)	1.5 to 4.3
Anterior, posterior, and distant organs	14 (2.8)	1.5 to 4.3
Anterior compartment	3 (0.6)	0.1 to 1.3
**Pain**		
Yes	473 (93.3)	91.0 to 94.8
No	34 (6.7)	5.0 to 8.8
**Menstrual period**		
Present	283 (55.7)	51.4 to 59.9
Absent	225 (44.3)	40.0 to 48.6
**Pain during menstrual period**		
Yes	268 (53.0)	48.7 to 57.3
No	14 (2.8)	1.5 to 4.3
Not menstruating at the moment	225 (44.3)	40.0 to 48.6
**Previous surgery for endometriosis**		
No	336 (66.1)	61.8 to 70.2
Yes	172 (33.9)	29.8 to 38.2
**Use of medication for endometriosis**		
Yes	298 (58.7)	54.4 to 63.0
No	210 (41.3)	37.0 to 45.6
**Physical activity**		
Practitioner	266 (52.4)	48.1 to 56.7
Non-practitioner	242 (47.6)	43.3 to 51.9
**Duration of physical activity practice**		
Non-practitioner	242 (47.6)	43.3 to 51.9
More than six months	173 (34.1)	30.0 to 38.4
Up to 6 months	93 (18.3)	15.0 to 21.9

n: number of participants. 95%CI: 95% confidence interval.

### Cluster identification and model quality

All participants were eligible for cluster analysis. Clusters were labeled according to the SDT. Among the motivational regulations and self-determination model, cluster 1 presented high values for controlled regulations and low values for autonomous regulations; therefore, the cluster was named ‘More controlled behavior’. Conversely, cluster 2 presented high values for autonomous regulations and low values for controlled regulations, named ‘More autonomous behavior’. Concerning the basic psychological needs model, participants of cluster 1 presented an increased perception of frustration with their BPN. This cluster was named ‘Basic psychological needs more thwarted’. On the other hand, cluster 2 was named ‘Basic psychological needs more satisfied’ because participants presented an increased perception of satisfaction with BPN.

The silhouette coefficient for the motivational regulations and self-determination model was 0.50, indicating a good-quality model (the clusters are reasonably well-separated), and for the basic psychological needs model was 0.60, indicating a high-quality model (well-separated clusters). According to commonly accepted thresholds, values above 0.50 are considered indicative of well-formed clusters, with higher values reflecting greater separation between clusters and stronger cohesion within clusters. The self-determination index had the highest relative importance (1.0) in estimating the SDT model followed by intrinsic regulation (0.69), and identified regulation (0.59), while introjected regulation had minimal influence on cluster formation (0.02). Autonomy, competence, and relatedness were highly important in evaluating the last model (1.0, 0.94, and 0.92, respectively).

### Motivational profile by cluster

Table 2 presents the distribution of the participants and the mean scores of the motivational variables in the clusters. Cluster 1 (‘More controlled behavior’) comprised 47.2% of the sample in the motivational regulations and self-determination model. For the BPN model, cluster 1 (‘Basic psychological needs more thwarted’) included 41.9% of the sample.

**Table 2:** Mean scores of motivational variables in each cluster.

**Motivational regulations and** **self-determination model**	**Cluster 1** **More controlled behavior** **(n = 240)**	**Cluster 2** **More autonomous behavior** **(n = 268)**
	**Mean ± SD**	**Mean ± SD**
Amotivation	1.38 ± 0.95	0.21 ± 0.42
External regulation	1.57 ± 1.14	0.81 ± 0.91
Introjected regulation	1.64 ± 1.22	2.00 ± 1.14
Identified regulation	1.73 ± 1.00	3.35 ± 0.54
Integrated regulation	1.03 ± 0.88	2.69 ± 0.98
Intrinsic regulation	1.19 ± 0.89	3.08 ± 0.76
Self-determination index	−1.57 ± 4.80	13.7 ± 5.03
**BPN model**	**Cluster 1** **Basic psychological needs more thwarted** **(n = 213)**	**Cluster 2** **Basic psychological needs more satisfied** **(n = 295)**
	**Mean ± SD**	**Mean ± SD**
Autonomy	1.53 ± 0.57	3.43 ± 0.80
Competence	1.51 ± 0.50	3.27 ± 0.82
Relatedness	1.46 ± 0.70	3.60 ± 0.93

Cluster 1 (‘More controlled behavior’) shows higher amotivation and controlled regulations (external, introjected), whereas Cluster 2 (‘More autonomous behavior’) shows higher autonomous regulations (identified, integrated, intrinsic) and self-determination index.

BPN = basic psychological needs.

**Fig 1:**
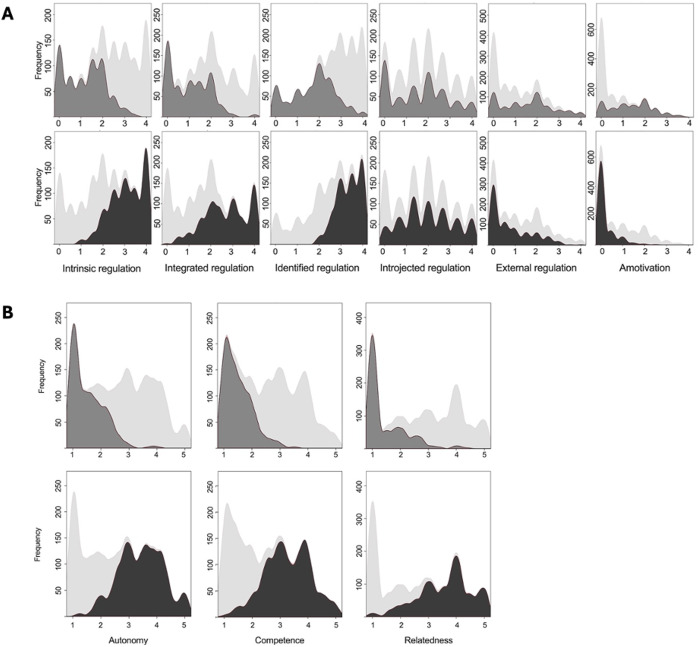
Cluster analysis of motivational regulations and basic psychological needs profiles. A) Motivational regulations profile based on cluster analysis. The upper panel (light grey) represents the mean scores of motivational regulations within the ‘More controlled behavior’ cluster, characterized by higher amotivation and external regulation scores. The bottom panel (dark grey) displays the mean scores of the ‘More autonomous behavior’ cluster, characterized by higher identified, integrated, and intrinsic regulation scores. The shaded area (very light grey) in both panels represent the distribution of scores for the full sample, providing a comparative reference. B) Basic psychological needs profile based on cluster analysis. The upper panel (light grey) represents the mean scores of the ‘Basic psychological needs more thwarted’ cluster, with lower autonomy, competence, and relatedness satisfaction. The bottom panel (dark grey) shows the ‘Basic psychological needs more satisfied’ cluster, which presented higher satisfaction in all three basic needs. The shaded area (very light grey) in both panels again illustrate the distribution of the full sample.

The ‘More controlled behavior’ cluster presented increased amotivation and external regulation and reduced identified, integrated, and intrinsic regulations and self-determination index compared with the ‘More autonomous behavior’ cluster. The self-determination index of the ‘More controlled behavior’ cluster and the ‘More controlled behavior’ cluster were −1.56 ± 4.80 and 13.7 ± 5.03, respectively. Regarding the BPN model, the ‘Basic psychological needs more thwarted’ cluster presented less autonomy, competence, and relatedness than the ‘Basic psychological needs more satisfied’ cluster (Fig 1).

### Clusters characterization

The mean age ranged between 33 and 34 years in both clusters. Pain score, marital status, time since diagnosis, physical activity participation, and participation in physical activity for more than six months are presented in Table 3.

**Table 3:** Sociodemographic and clinical characteristics of participants according to clusters.

	Motivational regulations and self-determination model		Basic psychological needs model	
	More controlled behavior	More autonomous behavior	Basic psychological needs more thwarted	Basic psychological needs more satisfied
**Age**	33.4 ± 7.3	34.2 ± 6.6	33.2 ± 6.7	34.2 ± 7.0
**Pain score**	7.7 ± 2.0	6.9 ± 2.4	8.0 ± 1.8	6.7 ± 2.4
**Marital status**				
With partner	143 (46.4%)	165 (53.6%)	125 (40.6%)	183 (59.4%)
Without partner	97 (48.5%)	103 (51.5%	88 (44.0%)	112 (56.0%)
**Skin color**				
White	130 (60.7)	183 (62.2)	102 (54.0)	211 (66.1)
Brown	61 (28.5)	63 (21.4)	55 (29.1)	69 (21.6)
Black	18 (8.4)	41 (13.9)	28 (14.8)	31 (9.7)
Yellow	4 (1.9)	6 (2.0)	2 (1.1)	8 (2.5)
Indigenous	1 (0.5)	1 (0.3)	2 (1.1)	0
**Educational level**				
Complete higher education	100 (46.7)	185 (62.9)	79 (41.8)	206 (64.6)
Incomplete higher education	47 (22.0)	52 (17.7)	39 (20.6)	60 (18.8)
High school	56 (26.2)	49 (16.7)	59 (31.2)	46 (14.4)
Complete middle school	5 (2.3)	7 (2.4)	7 (3.7)	5 (1.6)
Incomplete middle school	6 (2.8)	1 (0.3)	5 (2.6)	2 (0.6)
**Time since diagnosis**				
< 5 years	118 (49.8%)	119 (50.2%)	100 (45.2%)	137 (57.8%)
≥ 5 years	122 (45.0%)	149 (55.0%)	113 (41.7%)	158 (58.3%)
**Physical activity participation**				
Yes	58 (21.8%)	208 (78.2%)	38 (14.3%)	228 (85.7%)
No	182 (75.2%)	60 (24.8%)	175 (72.3%)	67 (27.7%)
**Physical activity for more than six months**				
Yes	27 (15.6%)	146 (84.4%)	21 (12.1%)	152 (87.9%)
No	31 (33.3%)	62 (66.7%)	17 (18.3%)	76 (81.7%)

Data are presented for Cluster 1 (‘More controlled behavior’ or ‘Basic psychological needs more thwarted’) and Cluster 2 (‘More autonomous behavior’ or ‘Basic psychological needs more satisfied’). The table allows comparison of demographic and clinical variables between clusters.

Age and pain scores are presented in mean ± standard deviation. The other variables are presented in absolute (relative) frequencies.

### Clusters and their association with physical activity participation

In the adjusted analysis, women from the ‘More controlled behavior’ (OR = 6.14; 95% CI = 3.72 to 10.1) and ‘Basic psychological needs more thwarted’ (OR = 7.61; 95% CI = 4.25 to 12.8) clusters presented reduced chance of participating in physical activity compared with those in the ‘More autonomous behavior’ and ‘Basic psychological needs more satisfied’ clusters, respectively (Table 4).

**Table 4:** Adjusted odds ratios for the association between motivational and basic psychological needs clusters and physical activity participation.

Variables^*^	Not participating in physical activity^#^			
	Crude		Adjusted	
	OR	95% CI	OR	95% CI
**Motivational regulations and self-determination model**				
More autonomous behavior cluster	1.00		1.00	
More controlled behavior cluster	10.88^*^	(7.20 to 16.4)	6.14^*^	(3.72 to 10.1)
**Basic psychological needs model**				
Basic psychological needs more satisfied cluster	1.00		1.00	
Basic psychological needs more thwarted cluster	15.67^*^	(10.05 to 24.4)	7.61^*^	(4.25 to 12.8)

Adjusted logistic regression model comparing the likelihood of physical activity participation across clusters. Women in the ‘More controlled behavior’ and ‘Basic psychological needs more thwarted’ clusters presented lower odds of participating in physical activity compared to those in the ‘More autonomous behavior’ and ‘Basic psychological needs more satisfied’ clusters, respectively. Values are presented as odds ratios (OR) with 95% confidence intervals (CI).

# Participating in physical activity is the reference group.

OR: Odds ratio; 95% CI: 95% confidence interval.

Models were adjusted for pain score, education level, skin color, and marital status.

* p < 0.01.

## Discussion

### Main findings

This study investigated the association between motivation clusters and physical activity participation in women with endometriosis, revealing two distinct behavioral patterns. One cluster exhibited more controlled behavior in the motivational regulations and self-determination model, whereas the other showed more autonomous behavior. Similarly, the BPN model identified a cluster with increased frustration and another with increased satisfaction with BPN. Thus, women with reduced motivational regulations and BPN support are six to seven times less likely to engage in physical activity.

In the present study, the motivational patterns aligned with SDT, suggesting that motivation may be improved with proper autonomy support and fulfillment of BPN. More autonomous approaches to motivation are related to better adaptative outcomes, such as behavior participation, enhanced performance, and improved well-being [17,22,34,35]. The ‘More controlled behavior’ cluster was mostly represented by amotivation and external regulation, highlighting that this cluster relied more on external than intrinsic factors. In contrast, the ‘More autonomous behavior’ showed integrated and intrinsic regulations and greater self-determination; thus, individuals in this cluster recognized more autonomous reasons (self-endorsement of the behavior, personal interest, satisfaction, and joy) for engaging in physical activity. Similarly, Kercher et al. [36] identified an “Autonomous-Focused” profile characterized by autonomous regulations and higher physical activity engagement, whereas a “Control-Focused” profile presented greater reliance on external motives (e.g., appearance and health concerns) and lower autonomy levels. The BPN model identified women experiencing higher frustration of their BPN for practicing physical activity. According to SDT, individuals who engage in behaviors without feeling a sense of choice, ownership, and competence demonstrate poorer performance quality, persistence, and well-being [14]. Therefore, women in the ‘More controlled behavior’ and ‘Basic psychological needs more thwarted’ clusters may find it challenging to engage in physical activity due to limited psychological interest.

The self-determination index emerged as the most influential factor in distinguishing clusters within the motivational regulations clustering model. This finding aligns with SDT, as the self-determination index effectively synthesizes the full continuum of motivational regulations, encapsulating both controlled and autonomous motivational behaviors [16]. Theoretically and mathematically, the self-determination index offered a comprehensive representation of motivational behavior, suggesting that it could serve as a reliable tool for screening. This metric holds promise not only for distinguishing motivational profiles but also for identifying individuals who may benefit from targeted interventions based on their motivational regulation styles [16].

Additionally, in the basic psychological needs model, the relative importance values revealed that autonomy, competence, and relatedness each contributed similarly to the formation of the clusters. This suggests that the distinction between the clusters was not driven by a single psychological need but rather by the collective perception of satisfaction or frustration across all three needs. This finding is consistent with SDT’s core proposition that the basic psychological needs operate in tandem to influence motivational outcomes [16]. The interplay between these needs underscores the complexity of motivational behavior and highlights the importance of considering all dimensions of psychological needs when evaluating motivation in individuals [16].

Given these motivational patterns, it is important to contextualize our findings with evidence from other populations with chronic conditions. These findings partially align with those observed in other chronic conditions, such as breast cancer and chronic pain, where patients similarly reported reduced autonomous motivation and increased controlled regulation, negatively influencing their physical activity engagement [5,37].

Additionally, when compared to broader literature, self-determination in women with endometriosis from the ‘More autonomous behavior’ cluster appears lower than that of healthy individuals of a similar age [38,39], resembling levels observed in impaired or older populations [36,37,40,41]. Furthermore, previous studies did not evaluate the motivational profile as lower as described in the ‘More controlled behavior’ cluster. Considering the similarities and contrasts between findings from previous studies, the present study provides a comprehensive understanding of motivational dynamics, particularly valuable in the absence of established cut-off points for the Behavioral Regulation in Exercise Questionnaire-3).

Young women with chronic conditions often experience high psychological distress due to persistent symptoms, uncertainty about disease progression, and potential limitations in daily life, amplifying emotional and social challenges [42,43], impacting their motivations and beliefs. Recently, there has been growing interest in psychosocial intervention, such as mindfulness and meditation, integrated into standard treatment plans to improve well-being in women with endometriosis [44].

Finally, the prolonged and often arduous diagnostic process for endometriosis, which can span years and involve significant physical and emotional distress [45,46], may exacerbate feelings of diminished control over one’s body. This lack of control can thwart the BPN, potentially spilling over into reduced motivation for exercise behaviors, as observed in the ‘Basic psychological needs more thwarted’ cluster. These findings underscore the importance of addressing psychological barriers in SDT-based interventions to enhance exercise engagement among women with endometriosis.

### Implications for practice

The difference between the two clusters underscores the crucial role of autonomous motivation and self-determination in shaping behavior, emphasizing the need for exercise and rehabilitation programs for women with endometriosis to include behavior change strategies tailored to specific motivational profiles [13], such as the ‘More controlled behavior’ and ‘Basic psychological needs more thwarted’ clusters.

Behavior change techniques are broadly applicable [17]. Women in the ‘More controlled behavior’ or ‘Basic psychological needs more thwarted’ clusters often lack the personal desire or will for physical activity, relying only on external pressures as their motivation [19] which hinders both initiation and long-term adherence. For these individuals, interventions should prioritize autonomy-supportive strategies, such as providing meaningful rationales, addressing barriers, using non-controlling language, and gradually building competence [17,22]. Conversely, for women in the ‘More autonomous behavior’ or ‘Basic psychological needs more satisfied’ clusters, programs should focus on sustaining autonomous motivation through optimized goal setting, maintaining challenge and interest, and offering supportive feedback [16]. An example of practical implementation includes offering women in the ‘More controlled behavior’ cluster the opportunity to select from a variety of enjoyable activities, such as guided group walks or aerobic classes, where facilitators provide consistent positive feedback and acknowledge personal barriers [17,22]. For women in the ‘More autonomous behavior’ cluster, strategies may include collaboratively setting progressive goals, such as participating in local fitness challenges or peer-led sessions, aimed at sustaining interest and fostering a sense of mastery [16].

Recognizing better motivational qualities depends on participants experiencing competence, relatedness, and autonomy during physical activities [21]. Therefore, interventions directed to individuals with less self-determination and BPN support need a proper design to address these elements, creating an environment that encourages them to feel naturally self-directed, skilled, and connected [10,24,37,47,48]. Personalized SDT-based approaches are essential to reduce aversion, promote long-term engagement, and improve symptom management and quality of life [17,49].

However, practical barriers such as limited resources, healthcare providers’ lack of understanding of behavior change, and challenges integrating psychosocial support in clinical practice must be recognized [13]. Future strategies should address these barriers through professional education and organizational changes [49,50]. Rehabilitation professionals should be able to incorporate techniques that strengthen motivational elements into well-organized physical activity, exercise programs, or other rehabilitation services [13]. We also suggest the need for institutional support and interdisciplinary collaboration, following recommendations from recent literature [49,50].

Behavior change techniques based on SDT are known for their effectiveness in promoting behavior change in health contexts [17,22,35], presenting increased engagement correlating with improved motivation and behavior [21]. Examples of SDT strategies include providing choice, acknowledging perspectives, offering meaningful rationales, using noncontrolling language, presenting an intrinsic goal orientation, implementing behavioral practice, developing appropriate plans to the ability of each individual, emphasizing responsibility, exploring reasons, providing optimal challenges, offering informational feedback, providing support and encouragement, identifying barriers, fostering involvement, encouraging social support seeking, and cooperating with groups [13,17,26,49].

The findings highlight potential applications in broader public health campaigns and community-based exercise programs, promoting tailored interventions based on psychological profiles to enhance engagement and adherence at the population level [22]. Existing studies confirm the feasibility and effectiveness of SDT-based interventions across diverse populations, including those with chronic conditions, reinforcing the applicability of our results to clinical practice [17,49].

### Strengths and limitations

The present study was the first to highlight the usefulness of SDT for studying motivation for physical activity in women with endometriosis, revealing a significant contrast in exercise engagement between clusters. The findings exhibited the importance of motivational regulations and BPN support, providing insights into tailoring exercise participation and physical activity promotion in interventions. Future policies to enhance physical exercise among women with endometriosis could greatly benefit by integrating behavior change strategies into rehabilitation and exercise programs. These strategies, supporting BPN and recognizing self-determined reasons for exercise, embody a motivation-oriented rehabilitation approach, ensuring robustness and contextual relevance.

Despite the manuscript’s strengths and novel findings, such as its large sample size and robust statistical analysis, limitations should be acknowledged. I) The study employed online and snowball sampling methods that may have biased the sample toward women with access to endometriosis reference services, as all participants reported some follow-up care. This likely overrepresents digitally connected and health-engaged individuals, potentially skewing socioeconomic and geographic diversity and limiting generalizability to underserved populations. II) The reliance on self-reported data, including self-diagnosis of endometriosis in some regions with limited access to formal healthcare, and self-reported physical activity (level, type, and intensity), may have introduced biases. While this approach allowed for the inclusion of a broader and more diverse sample, it may limit the precision of the associations identified between motivational profiles and actual physical activity patterns. III) The cross-sectional design precludes understanding the longitudinal changes in motivation and physical activity patterns. Future longitudinal studies should explore how motivational profiles evolve over time, informing better-targeted interventions. This will enhance the robustness of findings, ultimately improving intervention strategies and outcomes for women with endometriosis. IV) We intentionally included the self-determination index as an independent variable in the cluster model to provide a more comprehensive understanding of an individual’s motivational profile. While the index is derived from the motivational regulations also in the model, potentially introducing some bias, its inclusion enhances the model’s ability to capture both the salience and overall perception of motivation. This approach allowed for a richer, more integrated description of motivational patterns.

## Conclusions

This inaugural study identified distinct motivational profiles associated with varying levels of self-determination and basic psychological needs satisfaction among women with endometriosis. Women with lower self-determination and BPN support were six to seven times less likely to engage in physical activity. These results emphasize the importance of shaping interventions that enhance autonomous motivation and support basic psychological needs by integrating behavior change techniques into exercise and rehabilitation programs for women with endometriosis, thereby improving engagement, adherence, and quality of life. Clinically, these findings highlight the importance of assessing motivational profiles and psychological needs when prescribing physical activity interventions for women with endometriosis.

The study’s novelty lies in applying SDT to identify motivational clusters specifically in women with endometriosis. Future longitudinal studies with more representative sampling and employing objective physical activity measurements are needed to reinforce these associations. Furthermore, investigations into effective SDT-based intervention components specifically for women with endometriosis represent critical unanswered questions that should be pursued.
